# International Clinical Trial Day and clinical trials in Ethiopia and Africa

**DOI:** 10.1186/1745-6215-15-493

**Published:** 2014-12-19

**Authors:** Abebaw Fekadu, Solomon Teferra, Asrat Hailu, Tsige Gebre-Mariam, Adamu Addissie, Wakgari Deressa, Getnet Yimer, Ahmed Reja

**Affiliations:** Department of Psychiatry, School of Medicine, College of Health Sciences, Addis Ababa University, Zambia Street, PO Box 9086, Addis Ababa, Ethiopia; Department of Psychological Medicine,, Centre for Affective Disorders, Institute of Psychiatry, King’s College London, DeCrespigny Park, SE5 8AF London, UK; Department of Microbiology, School of Medicine, College of Health Sciences, Addis Ababa University, Zambia Street, PO Box 9086, Addis Ababa, Ethiopia; Regional Bioequivalence Center, College of Health Sciences, Addis Ababa University, Zambia Street, PO Box 31708, Addis Ababa, Ethiopia; School of Public Health, College of Health Sciences, Addis Ababa University, Zambia Street, PO Box 9086, Addis Ababa, Ethiopia; Department of Pharmacology, School of Medicine, College of Health Sciences, Addis Ababa University, Zambia Street, PO Box 9086, Addis Ababa, Ethiopia; College of Health Sciences, Addis Ababa University, Zambia Street, PO Box 9086, Addis Ababa, Ethiopia

**Keywords:** Clinical trial, Developing country, Africa, Ethiopia, Trial registration

## Abstract

Low income countries like Ethiopia are underrepresented in clinical research. As a major public commitment to clinical research, Ethiopia celebrated the International Clinical Trial Day (ICTD) for the first time on 20 May 2014 under the auspices of Addis Ababa University. The motto for the day was ‘Clinical Trials for Excellence in Patient Care’. The celebration offered an opportunity to inform academic staff, researchers, students and the leadership about clinical trials being conducted and to discuss the future of clinical trials in the country. Although clear challenges to the conduct of trials abound, clinical trials registered from Ethiopia in trial registration databases is increasing. Cross-country collaborations, international funding support, motivation of academic staff to conduct clinical trials and the commitment and engagement of the leadership in research are all improving. The overall impact of clinical trials is also encouraging. For example, some of the trials conducted in Ethiopia have informed treatment guidelines. However, administrative capacity, research infrastructure as well as financial support remain weak. There is a need for enhanced university-industry linkage and translation of research findings into locally relevant evidence. Ethiopia, as well as the whole of Africa, has an unparalleled opportunity to lead the way in clinical trials, given its prospect of development and the need to have locally relevant evidence for its growing population. In this commentary we reflect on the celebration of ICTD, the status and opportunities for conducting clinical trials and the way forward for facilitating clinical trials in Ethiopia and Africa.

## Background

Ethiopia, with a population of over 90 million [1], is the second most populous country in Africa. Despite its tumultuous past, the country has shown potential for growth and change in recent years. For example, it is the second leading country in the world in terms of gains in life expectancy in the past two decades [2]. It has also made substantial gains in reducing maternal mortality [3] and child mortality [4]. The health system, focused on prevention and primary care, has also shown promising progress [5]. The number of medical schools has grown from three to over 30 in just two decades.

**Figure 1:**
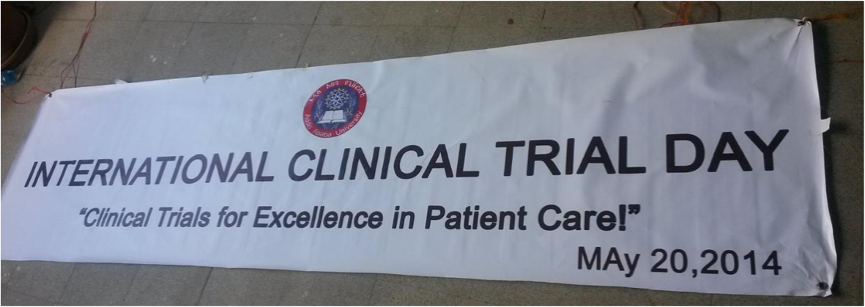
**Motto for the celebration of the International Clinical Trial Day, Addis Ababa, Ethiopia.**

The broad national indicators of progress happening in Ethiopia make it one of the most promising countries in Africa to conduct clinical trials. Within this context, Ethiopia celebrated its first International Clinical Trial Day (ICTD) on 20 May 2014, with the motto of ‘Clinical Trials for Excellence in Patient Care’ (Figure 1). The ICTD was hosted by the College of Health Sciences at Addis Ababa University, and was commemorated with various presentations of ongoing clinical trials within the college, ranging from landmark trials on visceral leishmaniasis [6, 7] to randomized trials of schizophrenia [8, 9]. The establishment of a state of the art bioequivalence laboratory, only the second in Africa, was also celebrated on this occasion. We took this opportunity to reflect on the conduct of clinical trials in Ethiopia and Africa [10], and we hoped that these reflections may have resonance for other low income countries, particularly in sub-Saharan Africa.

## Main text

### Clinical trials in Africa and Ethiopia

**Figure 2:**
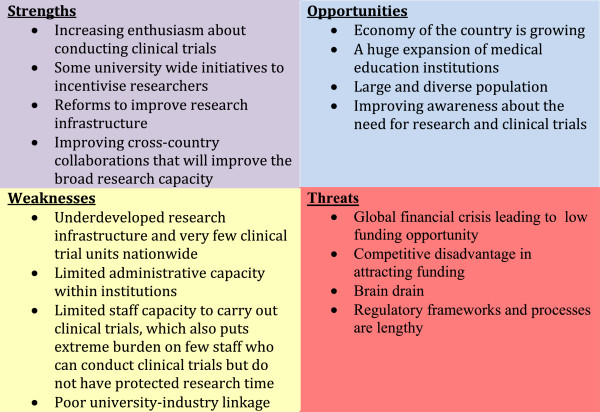
**Strength Weakness Opportunity and Threat (SWOT) analysis of the context for clinical trials in Ethiopia.**

**Figure 3:**
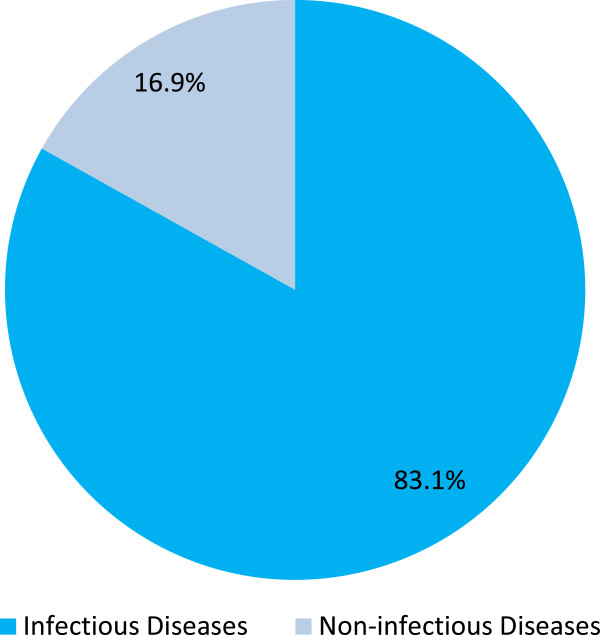
**Comparative proportion of trials for non-infectious and infectious diseases in Ethiopia (N = 59).** Based on data available at Clinicaltrials.gov (on 28 June 2014).

The global disparity in clinical research capacity and service provision may be indicated simply through the share of clinical trials a country or a region has registered in clinical trial registry databases. We have called this the ‘clinical trials index’ (CTI). For example, the share of studies registered from Africa (in Clinicaltrials.gov) is only 0.02%, although the region represents about 15% of the population of the world. Within Africa, most of the clinical trials are conducted in South Africa (47.3%), which makes one of the largest financial investments on health and has a relatively well-developed health system in Africa [11]. Only a limited number of clinical trials are registered from Ethiopia in trial registration sites, representing only 1.5% of all the studies from Africa. These CTI figures are crude reflections of the disparity in clinical research and service capacities within Africa and the world at large. Despite the limited number, some of the studies from Ethiopia were very important. For example, the World Health Organization (WHO) recommended sodium stibogluconate and paromomycin as first line treatments for visceral leishmaniasis in East Africa, based on clinical trials conducted in Ethiopia as part of an East African consortium (Leishmaniasis East Africa Platform (LEAP)) [12]. Equally important was the differential response pattern of leishmaniasis within the East African countries [12]. For example, in assessing the response to different formulations of stibogluconate, it was found that the response rate was lower among the Ethiopian sample [13]. Patients with HIV co-infection also had very poor treatment response [7]. Differential treatment response was also demonstrated between different regions within Ethiopia, partly accounted for by nutritional status [14]. This clearly underscores the need to have locally relevant evidence for different populations and the need for expertise in clinical trials in each country. Understandably, most of the clinical trials conducted in Ethiopia were for infectious diseases (Figures 2 and 3). However, clinical trials for non-communicable diseases (NCD) are also very important. First, NCDs are becoming increasingly relevant, and given their chronicity, are likely to take a big share of the health investment of any country. Underscoring the relevance of NCDs was a major focus on the United Nations Assembly in September 2011 [15]. NCDs have been considered a leading cause of death in all world regions with rapidly increasing burden [16]. In the context of the ‘double transition’, Africa is likely to be disproportionately affected by NCDs. Secondly, treatment of NCDs is likely to require more local adaptations than treatment of infectious conditions. Thirdly, the ethical requirements for the conduct of clinical trials for NCDs, particularly those that rely on non-medication interventions or those looking at secondary indications of existing treatments, are likely to be different.

### Ethics procedures in Ethiopia

Ethiopia has relatively advanced ethics procedures and review processes to protect participants in research. Various ethics committees are established at the level of institutions and there is one national committee. The national-level ethics committee, the National Research Ethics Review Committee (NRERC) [17], handles proposals with complex interventions and those with potentially higher risk to participants. All drug trials are also referred to the NRERC and drug trials are also regulated by the Food Medicine and Healthcare Authority of Ethiopia [18], which has the ultimate authority. The guidelines and terms of references of the ethics committees draw on international codes such as the Nuremberg Code, Belmont Report, the International Conference on Harmonization [19], Declaration of Helsinki and the Council for International Organizations of Medical Sciences. The Ethiopian Bioethics Initiative (ETBIN) was recognized as the first national chapter of the Pan-African Bioethics Initiative (PABIN). Its by-laws were recommended as templates for sister chapters in Africa [20].

### Challenges and opportunities of conducting clinical trials in Ethiopia and other African countries

**Figure 4:**
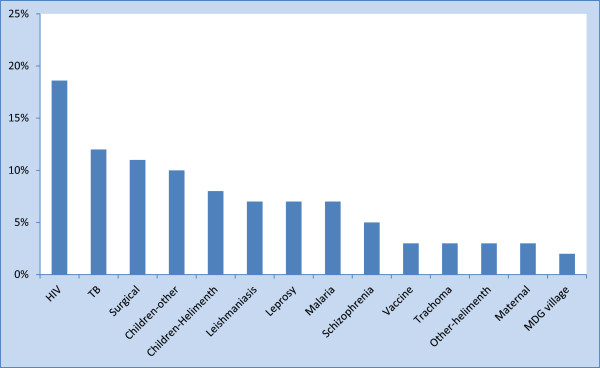
**Proportion of clinical trials conducted in specific subject or disease area in Ethiopia (N = 59).** Based on data available at Clinicaltrials.gov (on 28 June 2014).

A Strength, Weakness, Opportunity and Threat (SWOT) analysis matrix is presented in Figure 4. The challenges of conducting clinical trials in Ethiopia and other African countries partly relate to the unique requirements of clinical trials, but there are broader challenges that have applicability to conducting research in general. These include lack of appropriate infrastructure, for example space, supplies and maintenance; weak administrative capacity; lower prioritization of research in academic institutions given the need to produce a university educated work force; limited university-industry linkage; limited sources of funding; and the lack of equitable incentives for researchers.

Regulatory frameworks are either underdeveloped or imported directly without consideration of the local context. Regulatory functions are frequently slow and bureaucratic. However, plenty of opportunities exist for the conduct of clinical trials both in Ethiopia and other African countries. Research is receiving increasing recognition, particularly in some academic institutions; there is better networking and international collaboration, a massive expansion of medical schools and healthcare services, use of technology is improving and the generally improving economy in Ethiopia and many African countries are more conducive for conducting clinical research.

## Discussion

### The way forward

It is in Africa’s best interest to commit to clinical trials both to serve its people, its region and the world at large. Specific recommendations are listed below. Within Ethiopia, a recommendation was made to establish a clinical trial committee, which would conduct needs assessments, explore opportunities for collaboration, assess priority areas for clinical trials in line with the country’s priorities and make recommendations for effective establishment of leading clinical trial centers in the country. The main threats to conducting clinical trials are the poor administrative capacity in many institutions, the lack of expertise and brain drain to international institutions and non-governmental organizations (NGOs) within the country. Within country, brain drain occurs not only because of the financial incentives but because of the extremely limiting administrative capacity that hinders the conduct of clinical trials. Institutions and governments should address these needs urgently. Clinical trials should be grounded in solid ethical principles and should be conducted for the benefit of patients.

#### Recommendations on conducting clinical trials in low income settings

Clinical trials have the potential to benefit patients, institutions and nations if they are conducted with the right tools. Therefore all interested medical institutions should be encouraged to participate in the conduct of clinical trials. The following recommendations are meant to support institutional engagement in clinical trials:

1. Trials should be for patients’ benefit at all times. Competent regulatory frameworks should be able to scrutinize trials whether the principle of beneficence and non-maleficence are upheld. People in low income countries are particularly vulnerable owing to poverty, low education and restrained political empowerment. Therefore, stringent regulatory frameworks are required.
2. Even when regulatory frameworks have to be stringent, they also have to be efficient, especially in the context of international collaborations.
3. Both research and administrative capacity limitations are significant threats to the conduct of clinical trials. International collaborations should therefore support both administrative and research capacity development.
4. We would like to go even further. If international institutions or industries are interested in working with low income institutions in conducting clinical trials, they should consider it their ethical responsibility to support both human and material capacity development in low income countries before embarking on serious clinical trials.
5. Although the priority of clinical trials in low income countries will be on infectious diseases, it is important to understand that interventions (or the delivery mechanisms) for non-communicable disease require even more local contextualization. Considering this need for contextualization and the increasing burden they cause, it is not too early to focus attention on these diseases.
6. All institutions interested in participating in clinical trials should be encouraged to do so. Establishing institutional clinical trial committees may help to support capacity development, collaborations and setting priorities.
7. These institutions should also be prepared to be subject to regulatory standards set by local and international regulatory bodies. Full transparency and accountability in relation to registering trial protocols, monitoring and reporting are minimum standards.
8. Regulatory frameworks need to be contextualized for the country and treatment being considered.

Several measures to strengthen frameworks for the protection of patients, particularly the most vulnerable, in the area of clinical trials has been a main agenda of the government. Most institutions are committed to uphold these principles. The implementation of these principles in practice needs to be facilitated. We support full transparency in the conduct of clinical trials, including registration on web-based clinical trial registration databases. However, these regulatory frameworks have to be contextualized and relevant for the country and for the specific study [21]. Regulatory authorities should be supported to have the capacity and the confidence to make contextualized decisions, particularly in the context of international collaborations.

Finally, to the best of our knowledge, this is the first celebration of its kind in Africa. It might help for countries to celebrate the ICTD annually, both as an opportunity to celebrate clinical trials, and as an occasion to unite and push for the goal of improving the life of patients through clinical trials.

## Conclusions

Local evidence is essential and is more likely to influence policy and practice [22, 23]. There are some promising signs of progress in clinical research in Africa. For example, there is now the Pan-African Clinical Trials Registry [24], which is an important resource for clinical trials in Africa. The East African Consortium for Clinical Research [25], composed of five East African and five European countries, is another initiative that may support clinical trials in Africa. The number of clinical trials and investment in research is increasing. However, research in general, and clinical trials in particular lag far behind in Africa. This is demonstrated by the extremely low CTI. Attention should be paid to scaling up clinical trials, not only in infectious conditions, but also in NCDs. Strengthening regulatory frameworks to support researchers and protect participants should be encouraged. Establishing clinical trial units and human capacity development are essential ingredients to scaling up clinical trials in Africa. Researchers work in extremely difficult working environments, with limited support from the system. If clinical trials are to flourish, as they should, researchers have to be supported to focus on the delivery of clinical trials. Celebrating ICTD provides an important platform to create awareness and to advocate on behalf of clinical trials, researchers and patients.

## Abbreviations

CTI: Clinical Trials Index
ETBIN: Ethiopian Bioethics Initiative
HIV: Human Immunodeficiency Virus
ICTD: International Clinical Trial Day
LEAP: Leishmaniasis East African Platform
NCD: Non-Communicable Diseases
NGOs: Non-Governmental Organizations
NRERC: National Research Ethics Review Committee
PABIN: Pan-African Bioethics Initiative
SWOT: Strength, Weakness, Opportunity, Threat
WHO: World Health Organization.

## References

[1] **World Population Prospectus: The 2012 Revision Highlights and Advance Tables** [http://esa.un.org/wpp/documentation/pdf/WPP2012_HIGHLIGHTS.pdf]

[2] World Health Organization (2014). World Health Statistics.

[3] **Trends in Maternal Mortality: 1990 to 2013. Estimates by WHO, UNICEF, UNFPA, The World Bank and the United Nations Population Division** [http://apps.who.int/iris/bitstream/10665/112682/2/9789241507226_eng.pdf?ua=1]

[4] **Levels and trends in Child Mortality: Report 2013** [http://www.childinfo.org/files/Child_Mortality_Report_2013.pdf]

[5] Donnelly J (2011). Ethiopia gears up for more major health reforms. Lancet.

[6] Musa A, Khalil E, Hailu A, Olobo J, Balasegaram M, Omollo R, Edwards T, Rashid J, Mbui J, Musa B, Abuzaid AA, Ahmed O, Fadlalla A, El-Hassan A, Mueller M, Mucee G, Njoroge S, Manduku V, Mutuma G, Apadet L, Lodenyo H, Mutea D, Kirigi G, Yifru S, Mengistu G, Hurissa Z, Hailu W, Weldegebreal T, Tafes H, Mekonnen Y, Makonnen E, Ndegwa S, Sagaki P, Kimutai R, Kesusu J, Owiti R, Ellis S, Wasunna M (2012). Sodium Stibogluconate (SSG) & paromomycin combination compared to SSG for visceral leishmaniasis in East Africa: a randomised controlled trial. PLoS Negl Trop Dis.

[7] Diro E, Lynen L, Mohammed R, Boelaert M, Hailu A, van Griensven J (2014). High parasitological failure rate of visceral leishmaniasis to Sodium Stibogluconate among HIV Co-infected adults in Ethiopia. PLoS Negl Trop Dis.

[8] Fekadu A, Mesfin M, Medhin G, Alem A, Teferra S, Gebre-Eyesus T, Seboxa T, Assefa A, Hussien J, Lemma MT, Borba C, Henderson DC, Hanlon C, Shibre T (2013). Adjuvant therapy with minocycline for schizophrenia (The MINOS Trial): study protocol for a double-blind randomized placebo-controlled trial. Trials.

[9] Borba CPC, Fekadu A, Teferra S, Bekele D, Shibre T, Oppenheim CE, Schoenfeld DA, Henderson DC (2014). A placebo-controlled trial of folate with B12 in patients with schizophrenia with residual symptoms in Ethiopia using a sequential parallel comparison design. Br J Med Res.

[10] Sackett D (2013). Six pairs of things to celebrate on International clinical trials day. Trials.

[11] **World Health Organization: South Africa** [http://www.who.int/countries/zaf/en/]

[12] WHO Expert Committee (2010). Control of the Leishmaniasis: Report of a meeting of the WHO Expert Committee on the Control of Leishmaniases, 22–26 March 2010.

[13] Berman J (2006). Visceral leishmaniasis in the New World & Africa. Indian J Med Res.

[14] **Post-Kala-Azar dermal leishmaniasis: a manual for case management and control** [http://apps.who.int/iris/bitstream/10665/78608/1/9789241505215_eng.pdf]

[15] **Draft Political Declaration of the High-level Meeting on the prevention and control of non-communicable diseases** [http://www.un.org/en/ga/ncdmeeting2011/pdf/NCD_draft_political_declaration.pdf]

[16] Chan KY, Adeloye D, Grant L, Kolcic I, Marušic A (2012). How big is the next big thing? Estimating the burden of non–communicable diseases in low–and middle–income countries. J Global Health.

[17] **World Health Organization: National Bioethics Committees in the African Region** [http://www.who.int/ethics/committees/afro/en/]

[18] **Food, Medicine and Health Care Administration and Control Authority of Ethiopia** [http://www.fmhaca.gov.et/]

[19] **The International Conference on Harmonisation of Technical Requirements for Registration of Pharmaceuticals for Human Use** [http://ich.org/] 10.1111/j.1365-2125.1994.tb05705.x PMC1364893 8054244

[20] **Pan-African Bioethics Initiative** [http://www.who.int/sidcer/fora/en/pabin3rd.pdf]

[21] Lang T, Cheah PY, White NJ (2011). Clinical research: time for sensible global guidelines. Lancet.

[22] Franzen SRP, Chandler C, Enquselassie F, Siribaddana S, Atashili J, Angus B, Lang T (2013). Understanding the investigators: a qualitative study investigating the barriers and enablers to the implementation of local investigator-initiated clinical trials in Ethiopia. BMJ Open.

[23] Franzen SRP, Chandler C, Atashili J, Angus B, Lang T (2013). Barriers and enablers of locally led clinical trials in Ethiopia and Cameroon: a prospective, qualitative study. Lancet.

[24] **Pan-African Clinical Trials Registry** [http://www.pactr.org/]

[25] **East African Consortium for Clinical Research** [http://eaccr.org/]

